# Post-operative segmental cerebral venous sinus thrombosis: risk factors, clinical implications, and therapeutic considerations

**DOI:** 10.1007/s10143-023-02067-4

**Published:** 2023-07-03

**Authors:** Carmelo Lucio Sturiale, Anna Maria Auricchio, Iacopo Valente, Alessandro Vacca, Giovanni Pennisi, Gabriele Ciaffi, Alessio Albanese, Alessando Olivi, Gianluca Trevisi

**Affiliations:** 1 Department of Neurosurgery, Fondazione Policlinico Universitario A. Gemelli IRCCS, Università Cattolica del Sacro Cuore, L.go A. Gemelli, 8 – 00168 Rome, Italy; 2 Department of Neurosurgery, University of Helsinki and Helsinki University Hospital, Helsinki, Finland; 3 Department of Radiology, Fondazione Policlinico Universitario A. Gemelli IRCCS, Università Cattolica del Sacro Cuore, L.go A. Gemelli, 8 – 00168 Rome, Italy; 4 Department of Neurosciences, Imaging and Clinical Sciences, G. D’Annunzio University, Chieti-Pescara, Italy; 5 Neurosurgical Unit, Ospedale Spirito Santo, Pescara, Italy

**Keywords:** venous sinus, Silent thrombosis, Supratentorial and infratentorial craniotomy, Anticoagulants, Long-term outcome

## Abstract

Cerebral venous sinus thromboses (CVSTs) are rare complications of neurosurgical interventions and their management remains controversial as most of cases appear clinically silent. Here, we analyzed our institutional series of patients with CVSTs evaluating clinical and neuroradiological characteristics, risk factors, and outcome. From the analysis of our institutional PACS, we collected a total of 59 patients showing postoperative CVSTs after supratentorial or infratentorial craniotomies. For every patient, we collected demographics and relevant clinical and laboratory data. Details on thrombosis trend were retrieved and compared along the serial radiological assessment. A supratentorial craniotomy was performed in 57.6% of cases, an infratentorial in 37.3%, while the remaining were a single cases of trans-sphenoidal and neck surgery (1.7%, respectively). A sinus infiltration was present in almost a quarter of patients, and in 52.5% of cases the thrombosed sinus was exposed during the craniotomy. Radiological signs of CVST were evident in 32.2% of patients, but only 8.5% of them developed a hemorrhagic infarct. CVST-related symptoms were complained by 13 patients (22%), but these were minor symptoms in about 90%, and only 10% experienced hemiparesis or impaired consciousness. The majority of patients (78%) remained completely asymptomatic along the follow-up. Risk factors for symptoms occurrence were interruption of preoperative anticoagulants, infratentorial sinuses involvement and evidence of vasogenic edema and venous infarction. Overall, a good outcome defined mRS 0–2 was observed in about 88% of patients at follow-up. CVST is a complication of surgical approaches in proximity of dural venous sinuses. CVST usually does not show progression and courses uneventfully in the vast majority of cases. The systematic use of post-operative anticoagulants seems to not significantly influence its clinical and radiological outcome.

## Introduction

Besides to be a spontaneous event in several systemic conditions such as infection, malignancies, hematologic disorders, and trauma, cerebral venous sinus thromboses (CVST) can be also complications of neurosurgical interventions, especially in case of lesions or surgical approaches involving the dural sinuses with a reported incidence estimated between 5 and 12% [1–4].

CVSTs are generally reputed as severe complications because they represent potential life-threatening conditions. They are also considered very difficult to be managed, since the anticoagulant therapy, which is life-saving in these cases, can induce the hemorrhagic evolution of a CVST-related venous infarction.

However, post-surgical CVSTs frequently appear segmental and not progressive; therefore, apparently, they do not seem sharing the same natural history of the spontaneous forms, even though they can present similar pathophysiological mechanisms. In fact, postoperative CVSTs often remain clinically silent [4], and only a small percentage of patients become symptomatic depending on thrombosis extension and compensation effectiveness of the collateral venous drainage supply [1, 5].

In this paper, we analyze our institutional series of patients undergoing supra- and sub-tentorial craniotomies for various intracranial pathologies and evaluated the factors, neuroradiological and clinical characteristics and outcome associated with the development of a postoperative CVST.

## Materials and methods

### Data collections

Using the keyword “sinus thrombosis” we searched within the institutional PACS to identify all cases with a radiological diagnosis of CVST in the last 5 years. After a first review of all retrieved CT-angiography (CTA) and MR-angiography (MRA) studies, we selected only patients who had evidence of CVST occurrence following a recent neurosurgical intervention (within 4 weeks).

### Measurements

For each patient, we collected demographics and relevant clinical data such as diabetes, blood pressure, smocking habit, preoperative use of anti-thrombotics, and postoperative use of heparin. We also collected the most important laboratory data, particularly the main coagulation tests such as activated partial thromboplastin time (aPTT), international normalized ratio (INR), fibrinogen, and platelet cell count.

In all patients, details on pathology and surgical technique such as sinus exposure and/or bleeding during the craniotomy were specifically retrieved. Regarding the thrombosis, we collected data on its location, extension (in mm), related symptoms, and associated radiological picture such as brain swelling or intracerebral hematoma.

All patients underwent clinical and radiological follow-up with serial CTA or MRA. The post-operative protocol envisaged for all patients the execution of a CT- or MRI-scan study with contrast enhancement, supplemented by angiographic sequences in case of evidence of signs of obliteration of a venous sinus. All patients, except those for whom there was a specific contraindication, were subjected to a postoperative prophylactic regimen of low molecular weight heparin (LMWH) at a dosage of 4000 IU daily, starting on the first or second postoperative day usually after a neuroradiological screening to rule out any bleeding in the surgical cavity. On the other hand, in case of evidence of sinus thrombosis it was decided on individual basis whether to start a therapeutic dosage of LMWH (100 mg/kg twice daily) which was subsequently shifted to oral anticoagulant therapy for a period of weeks or months based on the subsequent angiographic controls.

Clinical outcome was recorded at discharge and at follow-up using the modified Ranking Scale (mRS). A good outcome was defined as mRS 0–2. The timing of radiological follow-up was set on individualized clinical needs, taking into account original pathology and presence of symptomatic postoperative CVST. Details on thrombosis trend were retrieved and compared along the radiological follow-up and classified as follows: resolved, persistent (improved or stable), and worsened. In case of complete resolution, date of the scan was considered as the thrombosis healing date. In case of thrombosis persistence or worsening, we recorded the date of last clinical-radiological follow-up.

### Statistical analysis

Descriptive statistics was used to label the frequency and mean value of the qualitative and quantitative data, respectively. Patients were divided into two groups according to presence or absence of thrombosis-related clinical signs. Quantitative variables were expressed as mean ± standard deviation and Student’s *t*-test was used to compare their means. Pearson’s Chi-Squared was instead used to compare categorical variables. Alpha was set at 0.05.

A linear regression model was built to verify the possible association between the extension in mm of the thrombosis and the following covariates: age, gender, aPTT, INR, fibrinogen value, platelet cells count, preoperative use of anticoagulants, or antiplatelet drugs, postoperative use of anticoagulants.

A Cox proportional hazards regression analysis was used to assess all the retrieved demographic, clinical and pathological data as possible risk factors of non-healing of the thrombosis at follow-up scans.

Jamovi 2.3.24 open-source software was used for statistical analysis.

## Results

We collected 59 patients showing postoperative CVST after supratentorial or infratentorial craniotomies. Demographic, clinical, and radiological data regarding the entire population are shown in Table 1. The mean age was 54.3 ± 17.0 years, with a slight prevalence of females (34; 58%). Regarding risk factors, 36 patients (61%) had arterial hypertension, 8 (14%) diabetes, and about a quarter (14; 24%) smoke habits.

**Table 1:** Demographic, clinical, and radiological data

Total number of patients		59 (%)
Age in years (mean±st.dev.)		54.3 ± 17.0
Female gender		34 (58)
Diabetes		8 (14)
High blood pressure		36 (61)
Smoking habit		14 (24)
Mean aPTT (mean±st.dev.)		31.0 ± 5.2
INR (mean±st.dev.)		1.1 ± 0.1
Fibrinogen (mg/dL) (mean±st.dev.)		334.7 ± 149.8
Platelet count (*10^9^/L) (mean±st.dev.)		263.6 ± 89.5
On preoperative antiplatelets		8 (14)
On preoperative anticoagulants		7 (12)
Pathology	Glioma	5 (8.5)
	Inflammatory	4 (6.8)
	Meningioma	14 (23.7)
	Metastasis	8 (13.6)
	Post-traumatic hematoma	7 (11.9)
	Schwannoma	10 (16.9)
	Vascular malformation	6 (10.2)
	Other	5 (8.5)
Side of pathology	Right	24 (40.7)
	Left	33 (55.9)
	Bilateral	2 (3.4)
Location of Pathology	Supratentorial	34 (57.6)
	Sella	1 (1.7)
	C-P angle	16 (27.1)
	Cerebellar	6 (10.2)
	Brainstem	1 (1.7)
	Neck	1 (1.7)
Sinus infiltration		14 (24)
Sinus exposure with craniotomy		31 (52.5)
Sinus bleeding during surgery		16 (27)
Location of thrombosis	Sovratentorial	17 (28.8)
	Subtentorial	38 (64.4)
	Sovra- and sub-tentorial	4 (6.8)
Side of thrombosis	Right	18 (30.5)
	Left	24 (40.7)
	Midline	17 (28.8)
Thrombosis extension in mm (mean±st.dev.)		31.7 ± 13.6

Abbreviations: *aPTT* activated partial thromboplastin time, *INR* international normalized ratio, *C-P Angle* cerebello-pontine angle

Coagulation lab tests and platelets count revealed on average normal values. In the preoperative period, 8 patients (14%) were under antiplatelet medications, and 7 (12%) under anticoagulants. The first category usually withdraws the medications on average 7 days before surgery, whereas the second stopped the anticoagulants at least 3–4 days before surgery until having a renormalization of the lab tests the day before surgery and is subjected to a preoperative prophylactic regimen of low molecular weight heparin.

Most of patients developing a post-surgical CVST underwent craniotomy for meningiomas removal (14; 23.7%) and cerebello-pontine angle schwannomas (10; 16.9%); less often for metastasis (8; 13.6%), traumatic hematomas (7; 11.9%), vascular malformations (6; 10.2%), gliomas (5; 8.5%), and inflammatory conditions (4; 6.8%). Just over half of the patients underwent a supratentorial craniotomy (34; 57.6%), while a trans-sphenoidal approach and a neck surgery were observed in single cases (1.7% respectively). The remaining cases of CVST were reported after infratentorial craniotomies. In particular: 16 patients (27.1%) underwent a surgical procedure on the cerebellar-pontine angle, 6 patients (10.2%) a suboccipital midline craniotomy, and 1 a far-lateral approach for a brainstem lesion.

We also assessed the presence of technical risk factors during the approach potentially associated with CVST observing that in almost a quarter of patients the original lesion showed a sinus infiltration (mostly meningiomas), but in all cases a residual patency of the sinus was demonstrated at the preoperative assessment with angio-MRI in venous phase, in 31 cases (52.5%) the thrombosed sinus was exposed by the craniotomy, and in 16 (27%) a minimal sinus bleeding was described during the craniotomy, which was controlled with gelfoam and pressure, and no suturing of the sinus wall was ever required.

A midline CVST involving the superior sagittal sinus (SSS) was observed in 17 supratentorial procedures (28.8%), while the involvement of an infratentorial venous hemi-system including transvers sinus / sigmoidal sinus/jugular vein was observed in 42 patients, 18 (30.5%) on the right side and 24 (40.7%) on the left side. The extension of the sinus thrombosis was on average 31.7 ± 13.6 mm. Frequencies and percentages of sinus involvement are reported in Table 2.

**Table 2:** Frequencies for involved sinus

Involved Sinus	Frequency	Percentage
Superior sagittal sinus	16	27.1
Transverse sinus	1	1.7
Sigmoid sinus	5	8.5
Jugular vein	3	5.1
Transverse sinus + sigmoid sinus	13	22.0
Transverse sinus + sigmoid sinus + jugular vein	11	18.6
Sigmoid sinus + jugular vein	6	10.2
Superior sagittal sinus + transverse sinus + sigmoid sinus + jugular vein	2	3.4
Superior sagittal sinus + transverse sinus + sigmoid sinus	1	1.7
Superior sagittal sinus + straight sinus	1	1.7
Total	59	100

At post-operative neuroradiological examination, signs of CVST were evident in 19 patients (32.2%). In particular, 14 (23.7%) showed vasogenic edema from venous congestion, while 5 (8.5%) a hemorrhagic infarct. Overall, 13 patients (22%) complained CVST-related symptoms, which were mostly mild such as headache (6 cases; 10.2%) and a single case of seizures (1.7%). Severe symptoms were instead recorded in about 10.2% of cases: among them, 5 patients (8.5%) experienced hemiparesis, and 1 (1.7%) impairing of state of consciousness after a normal recovery from anesthesia. No association was seen between side of thrombosis and clinical signs. This lack of association was confirmed also including the presence of a dominant transverse-sigmoid hemi-system in the statistics. Namely, no difference in clinical course was seen if thrombosis occurred in the dominant or non-dominant hemi-system.

However, thrombosis-related symptoms where significantly more frequent when SSS, alone or together with other sinuses, was involved (8/20 cases, 40%), compared with 5/39 (12.8%) patients who complained symptoms after a thrombosis in other sinuses (transverse/sigmoid/jugular, alone or in combination), *p* = 0.01.

Considering the location of SSS thrombosis, no significant difference in development of symptoms was seen. Namely, anterior SSS alone was involved in 8 cases (3 with symptoms), median third of SSS in 6 cases (2 with symptoms), posterior SSS in 2 cases (none with symptoms), and, as reported in Table 2, an extensive thrombosis of the SSS extended to other sinuses in 4 cases (3 with symptoms).

On the other hand, the majority of patients (46 out of 59; 78%) remained completely asymptomatic (Table 3).

**Table 3:** Frequency of thrombosis-related clinical symptoms and radiological lesions

Total n. of pts = 59		*n* (%)
Radiological lesions	Intracerebral hemorrhage	5 (8.5)
	Edema	14 (23.7)
Clinical symptoms	Asymptomatic	46 (78)
	Decreased consciousness	1 (1.7)
	Headache	6 (10.2)
	Hemiparesis	5 (8.5)
	Seizures	1 (1.7)

A good clinical outcome at discharge (mRS 0–2) was observed in 48 patients (81.4%), whereas a poor outcome (mRS 3–6) in 11 (18.6%) with a mRS on average of 1.47 ± 1.42. At mean clinical and radiological follow-up of 20.8 ± 19.0 months, the number of patients in good outcome (mRS 0–2) was overall 52 (88.1%), whereas those in poor outcome were 7 (11.9%) with a mean mRS of 0.8 ± 1.5 (Table 4).

**Table 4:** Clinical and radiological outcome at discharge and follow-up

Total number of patients		59 (%)
Patients with thrombosis related symptoms		13 (22)
Mean modified Rankin Scale (mRS) at discharge (mean±st.dev.)		1.47 ± 1.42
Modified Rankin Scale groups at discharge	mRS 0	16 (27.1)
	mRS 1	19 (32.2)
	mRS 2	13 (22)
	mRS 3	6 (10.2)
	mRS 4	3 (5.1)
	mRS 5	0
	mRS 6	2 (3.4)
Mean follow-up in months (mean±st.dev.)		20.8 ± 19.0
Mean modified Rankin Scale (mRS) at follow-up (mean±st.dev.)		0.8 ±1.5
Modified Rankin Scale groups at follow-up	mRS 0	39 (66.1)
	mRS 1	10 (16.9)
	mRS 2	3 (5.1)
	mRS 3	4 (6.8)
	mRS 4	0
	mRS 5	0
	mRS 6	3 (5.1)
Persistence of thrombosis at follow-up		36 (61)
Mean time for thrombosis healing in months (mean±st.dev.)		14.5 ± 13.0

Overall, 39 patients (66%) were put on therapeutic LMWH dosage, including all the 13 symptomatic patients.

The choice of starting therapeutic anticoagulation was non based on the involved sinus, despite a non-significant more frequent use of therapeutic LMWH when SSS was involved (15/5, 75% Vs. 24/15, 61.5%; *p* = 0.3). All 5 cases who developed an ICH were among the 39 patients on therapeutic LMWH dosage; however, this correlation was not statistically significant (*p* = 0.09). However, no difference in clinical outcome was seen between patients on therapeutic LMWH and patients who were not put on therapeutic anticoagulation at both discharge (mRS 1.7 ± 1.5 Vs. 1 ± 1, respectively; *p* = 0.06) and follow-up (mRS 0.8 ± 1.5 Vs 0.65 ± 1.5, respectively; *p* = 0.6).

From a radiological point of view, a residual thrombosis was still observed in 36 patients (61%) at last clinic-radiological follow-up. In the remaining 28 cases where a complete healing was observed at neuroimaging, the mean time of healing was 14.5 ± 13.0 months. Curiously, patients on therapeutic anticoagulation took longer to completely heal compared to those who were not put on therapeutic anticoagulation (17.9 ± 14.5 months vs. 7.1 ± 3.3 months). However, this difference was not statistically significant (*p* = 0.07) (Table 4).

By comparing symptomatic and asymptomatic patients (13 versus 46) it was found that risk factors for symptoms occurrence were interruption of preoperative anticoagulants, sovra- and sub-tentorial sinuses involvement, and appearance of vasogenic edema and/or venous infarction (Table 5).

**Table 5:** Variables associated with thrombosis related clinical signs

Total number of patients = 59		Asymptomatic *n* = 46 (78%)	Symptomatic *n* = 13 (22%)	Tota l *n* (%)	*p*-value
Age (mean±st.dev.)		54.9 ±18.6	52.5 ±21.3		0.69
Female gender		24 (52%)	10 (77%)	34 (58%)	0.20
Diabetes		6 (13%)	2 (15%)	8 (14%)	1.00
High blood pressure		26 (56.5%)	10 (77%)	36 (61%)	0.21
Smoking habit		12 (26%)	2 (15%)	14 (24%)	0.71
aPTT (sec) (mean±st.dev.)		30.46 ± 5.2	32.85 ± 4.9	31.0 ± 5.2	0.81
INR (mean±st.dev.)		1.06 ± 0.1	1.08 ± 0.1	1.1 ± 0.1	0.92
Fibrinogen (mg/dL) (mean±st.dev.)		315.85 ± 130.7	401.62 ± 195.3	334.7 ± 149.8	0.75
Platelet count (*10^9^/L) (mean±st.dev.)		263.26 ± 90.8	265 ± 88	263.6 ± 89.5	0.99
On preoperative antiplatelets		7 (15%)	1 (8%)	8 (14%)	0.67
On preoperative anticoagulants		3 (6.5%)	4 (31%)	7 (12%)	**0.01**
Pathology	Glioma	5 (10.9%)	0	5 (8.5%)	0.57
	Inflammatory	4 (8.7%)	0	4 (6.8%)	0.56
	Meningioma	10 (21.7%)	4 (30.8%)	14 (23.7%)	0.48
	Metastasis	6 (13%)	2 (15.4%)	8 (13.6%)	1.00
	Post-traumatic hematoma	4 (8.7%)	3 (23.1%)	7 (11.9%)	0.17
	Schwannoma	9 (19.6%)	1 (7.7%)	10 (16.9)	0.43
	Vascular malformation	4 (8.7%)	2 (15.4%)	6 (10.2%)	0.60
	Other	4 (8.7%)	1 (7.7%)	5 (8.5%)	1.00
Location of thrombosis	Sovratentorial	12 (26%)	5 (38.5%)	17 (28.8%)	0.49
	Subtentorial	33 (72%)	5 (38.5)	38 (64.4%)	**0.02**
	Sovra- and sub-tentorial	1 (2%)	3 (23%)	4 (6.8%)	**0.008**
Sinus infiltration		10 (22%)	4 (31%)	14 (24%)	0.48
Sinus exposure with craniotomy		22 (48%)	9 (70%)	31 (52.5%)	0.21
Sinus bleeding during surgery		12 (26.1%)	4 (30.8%)	16 (27%)	0.73
Extension of thrombosis in mm (mean±st.dev.)		30.22 ± 12.9	37.15 ± 15.2	31.7 ± 13.6	0.79
Thrombosis-related intracerebral bleeding		0	5 (38.5%)	5 (8.5%)	**<.001**
Thrombosis-related edema		1 (2.2%)	13 (100%)	13 (24%)	**<.001**

Significant *p*-values are highlighted in bold

A multivariate model exploring the association between thrombosis extension and demographic and pharmacological variables did not confirm any independent risk association with preoperative lab-test values and anticoagulants/antiplatelets assumption (Table 6).

**Table 6:** Association between the extension of the thrombosis and demographic and pharmacological data

Covariates	Unstandardized	Standard error	Standardized^a^	t	*p*-valu**e**
Age	−0.108	0.115	−0.134	−0.937	0.353
aPTT (sec)	−0.535	0.425	−0.202	−1.258	0.214
INR	29.725	20.305	0.234	1.464	0.150
Fibrinogen (mg/dL)	0.006	0.013	0.061	0.429	0.670
Platelet count (*10^9^/L)	−0.020	0.021	−0.129	−0.918	0.363
Female gender	1.076	3.894	-	0.276	0.784
Preoperative anticoagulants	4.622	5.993	-	0.771	0.444
Preoperative antiplatelets	−3.021	5.496	-	−0.550	0.585
Postoperative Anticoagulants	3.488	4.035	-	0.864	0.392

^a^Standardized coefficients can only be computed for continuous predictors

**Fig. 1:**
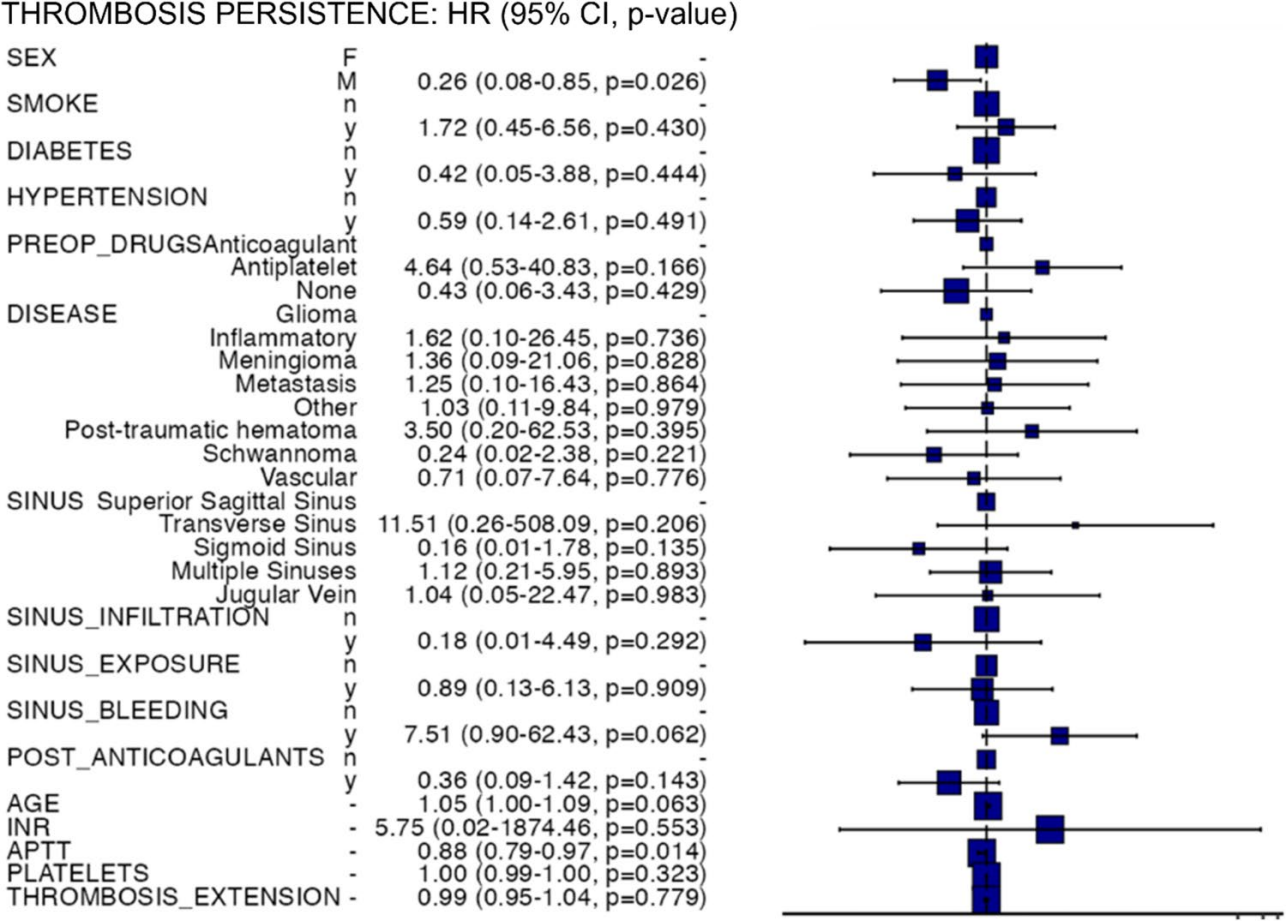
Hazards regression plot

Finally, we performed a Cox proportional hazards regression analysis to explore the risk of thrombosis persistence over time. At the multivariate model, only female sex and altered preoperative aPTT still appeared associated with risk of CVST persistence at follow-up. Noteworthy, the use of post-operative low molecular weight heparins seemed not influencing neither the thrombosis persistence nor the outcome (Table 7; Fig. 1).

**Table 7:** Risk of thrombosis persistence at follow-up

Dependent variable		All	Univariate model		Multivariate model	
			HR	*p*-value	HR	*p*-value
Sex	F	34 (100.0)	-	0.883	-	**0.026**
	M	25 (100.0)	1.05 (0.53–2.09)		0.26 (0.08–0.85)	
Smoke	N	45 (100.0)	-	0.616	-	0.430
	Y	14 (100.0)	1.21 (0.57–2.59)		1.72 (0.45–6.56)	
Diabetes	N	51 (100.0)	-	0.248	-	0.444
	Y	8 (100.0)	1.76 (0.67–4.62)		0.42 (0.05–3.88)	
Hypertension	N	23 (100.0)	-	0.769	-	0.491
	Y	36 (100.0)	1.11 (0.56–2.17)		0.59 (0.14–2.61)	
Preop_Drugs intake	Anticoagulant	7 (100.0)	-		-	
	Antiplatelet	8 (100.0)	4.58 (1.09–19.17)	**0.037**	4.64 (0.53–40.83)	0.166
	None	44 (100.0)	1.76 (0.53–5.85)	0.355	0.43 (0.06–3.43)	0.429
Disease	Glioma	5 (100.0)	-		-	
	Inflammatory	4 (100.0)	0.56 (0.11–2.84)	0.480	1.62 (0.10–26.45)	0.736
	Meningioma	14 (100.0)	0.56 (0.15–2.13)	0.395	1.36 (0.09–21.06)	0.828
	Metastasis	8 (100.0)	0.91 (0.23–3.58)	0.895	1.25 (0.10–16.43)	0.864
	Other	5 (100.0)	0.40 (0.08–2.04)	0.270	1.03 (0.11–9.84)	0.979
	Post-traumatic hematoma	7 (100.0)	0.91 (0.22–3.84)	0.899	3.50 (0.20–62.53)	0.395
	Schwannoma	10 (100.0)	0.16 (0.03–1.00)	0.050	0.24 (0.02–2.38)	0.221
	Vascular	6 (100.0)	0.59 (0.12–2.82)	0.511	0.71 (0.07–7.64)	0.776
Sinus involvement by the thrombosis	Superior sagittal sinus	16 (100.0)	-		-	
	Transverse sinus	1 (100.0)	3.96 (0.48–32.63)	0.201	11.51 (0.26–508.09)	0.206
	Sigmoid sinus	5 (100.0)	0.53 (0.12–2.41)	0.412	0.16 (0.01–1.78)	0.135
	Multiple sinuses	34 (100.0)	0.68 (0.33–1.40)	0.296	1.12 (0.21–5.95)	0.893
	Jugular vein	3 (100.0)	0.32 (0.04–2.49)	0.276	1.04 (0.05–22.47)	0.983
Sinus infiltration by the tumor	N	45 (100.0)	-		-	0.292
	Y	14 (100.0)	0.90 (0.42–1.93)	0.788	0.18 (0.01–4.49)	
Sinus exposure at surgery	N	28 (100.0)	-		-	0.909
	Y	31 (100.0)	1.21 (0.63–2.35)	0.564	0.89 (0.13–6.13)	
Sinus bleeding at surgery	N	43 (100.0)	-		-	0.062
	Y	16 (100.0)	1.57 (0.78–3.16)	0.207	7.51 (0.90–62.43)	
Post-operative anticoagulants	N	20 (100.0)	-	0.406	-	0.143
	Y	39 (100.0)	0.74 (0.37–1.50)		0.36 (0.09–1.42)	
Age	Mean (SD)	54.3 (17.0)	1.02 (1.00–1.04)	0.057	1.05 (1.00–1.09)	0.063
INR	Mean (SD)	1.1 (0.1)	0.11 (0.00–3.18)	0.201	5.75 (0.02–1874.46)	0.553
APTT	Mean (SD)	31.0 (5.2)	0.96 (0.90–1.02)	0.158	0.88 (0.79–0.97)	**0.014**
Platelets count	Mean (SD)	263.6 (89.5)	1.00 (0.99–1.00)	0.191	1.00 (0.99–1.00)	0.323
Thrombosis extension in mm	Mean (SD)	31.7 (13.6)	1.00 (0.97–1.02)	0.692	0.99 (0.95–1.04)	0.779

Model metrics: number in dataframe = 59, number in model = 59, missing = 0, number of events = 36, concordance = 0.785 (SE = 0.054), R-squared = 0.479 (Max possible = 0.983), likelihood ratio test = 38.522 (df = 26, *p* = 0.054)

Significant *p*-values are highlighted in bold

## Discussion

Our study confirmed that the natural history of post-surgical CVSTs is benign in the majority of cases. Up to 78% of patients showed a completely asymptomatic course, probably due to an effective compensation by the collateral venous system, while approximately 12% had minor clinical manifestations such as headache (10.2%) and self-limiting seizures (1.7%). Only a minority of patients (10.2%) showed severe complications such as hemiparesis (8.5%) and loss of consciousness (1.7%) after a normal recovery from anesthesia. On the other hand, radiological signs such as vasogenic edema or intracerebral hemorrhage were overall revealed in 32.2% of patients. Hence, up to 20% of patients showing radiological alterations continued to be asymptomatic over time and at the mean follow-up of more than 20 months, almost 90% of patients presented a good outcome (mRS ≤ 2). Moreover, among the 7 cases with a poor outcome (mRS > 2) at follow-up, 5 had a prognosis significantly influenced by the underlying pathology: in fact, 3 had a post-traumatic hematoma, and 2 a cerebral metastasis associated with a systemic progression of the oncological disease.

Notably, clinical symptoms were less frequent in cases with infratentorial venous sinuses involvement, despite presumably due to compensation by the contralateral venous system (Table 5).

Also, patients who were under anticoagulants before surgery for different reasons, mostly for atrial fibrillation, and temporarily interrupted the treatment just before the procedure, seemed to be at increased risk for symptomatic post-surgical CVST. This could represent a rebound effect, but its pathophysiological explanation still remains to be elucidated.

Surprisingly, the extension of the thrombosis did not appear influenced neither by the values of the preoperative laboratory coagulation tests, nor related to the occurrence of symptoms. Instead, the radiological persistence of the thrombus over time was more frequent in female patients with altered aPTT and did not appear influenced by the post-operative heparinization.

Typically, symptoms arise when venous outflow obstruction leads to increased intracranial pressure; therefore, headache is the most common presenting symptom. Other symptoms include nausea with or without emesis, altered mental status, visual changes, seizures, pseudotumor cerebri, and less commonly, focal neurological deficit [3, 5, 6]. More severe symptoms arise when CVST leads to a hemorrhagic stroke, which can be catastrophic [7].

### Infratentorial CVST

Patients undergoing posterior fossa surgery for cerebellopontine angle tumors are largely retained at increased risk for the development of CVST [3, 4, 8, 9].

Previous studies have documented rate of CVST between 4.7 and 11.6% among patients undergoing posterior fossa surgery [3, 6, 9]. However, other recent studies have shown that the rate of venous thrombosis after posterior fossa surgery is even greater when reviewed in a retrospective manner [10]. Brahimaj et al., in particular, reported about 35% of CVST incidence after vestibular schwannoma surgery [11]. It remains uncertain the reason why the majority of CVST are not identified on initial postoperative imaging. Indeed, CVST diagnosis appears underestimated on the initial reading of these cases, perhaps remaining unrecognized and not adequately considered among all postoperative changes at the surgical site, without dedicated vascular imaging.

Most of the patients reported in these series showed a postoperative lateral CVST on the same side of the craniotomy, and the transverse and sigmoid sinuses were the most commonly involved structures. However, these radiological pictures appeared very rarely associated to clinical symptoms [8].

This “clinical tolerance” of postoperative lateral CVST may be attributed to the presence of collateral venous channels or a contralateral dominant sinus. Moreover, in most patients, a postoperative CVST may be in general an inherently less aggressive pathological process than spontaneous CVST. In fact, on the contrary of the post-surgical CVST, the spontaneous sinus thrombosis is usually composed only of patients who have developed sufficiently severe symptoms to seek medical care. Nevertheless, surgeons should maintain a high index of suspicion after posterior fossa surgery to avoid missing postoperative CVST, particularly in asymptomatic patients, and a routine postoperative screening CT with angiograms is also recommended by some authors [8].

As regards to technical risk factors, interestingly, in the study by Abou-Al-Shaar et al., the degree of sinus exposure during craniotomy appeared inversely related to the risk of CVST. In fact, they found a slightly higher incidence of CVST after the retrosigmoid approach with partial sinus exposure (10%) compared with that after the translabyrinthine approach with complete sinus exposure (8%) [8].

Another interesting observation of some Authors is that in case of lateral CVST, the risk appears significantly higher in a co-dominant or nondominant sinus but not in a dominant sinus [8]. This may be related to a higher blood flow in the dominant sinus in comparison with nondominant and superficial venous drainage patterns. Higher throughput of blood in the dominant side may decrease the risk for venous stasis, thrombus formation, and thrombus propagation postoperatively. However, our data did not confirm the same findings.

In addition to mechanical sinus injury or accidental sinus opening or intentional debulking of intra-sinus lesions, several other factors were reported as able to cause CVST. Among them, the most important is the direct heat conduction during bipolar or monopolar coagulation, bony drilling, and thermal radiation from the operative microscope lamp as well as an excessive dural desiccation during the procedure [3, 6, 8, 9, 12].

Finally, the majority of studies on this topic do not include patient’s position during surgery as a major risk factor for CVST. As regards the posterior fossa surgery, in particular, in our series, the risk of a symptomatic CVST did not seem to be different between prone position and park bench position.

### Supratentorial CVST

Thrombosis of the SSS has been rarely reported after supratentorial surgery. In 1990, Garrido et al. described a case of cortical veins and SSS thrombosis after a transcallosal removal of a colloid cyst of the third ventricle, which hesitated in a poor prognosis [13].

More recently, Jimenez et al. reported a large series of patients treated for parasagittal/parafalcine meningiomas presenting with post-surgical SSS thrombosis. They reported about 5.6% of CVST incidence, but symptoms in only 1.9% of patients [14]. However, our case series collected through a careful retrospective radiological review also of the asymptomatic cases shows that supratentorial CVST is not so rare. In fact, we detected isolated SSS thrombosis in about 27% of all post-surgical CVST.

Occasionally, cases of SSS or cavernous sinus thrombosis were reported also after posterior fossa surgery with similar fatal outcome [15, 16]. However, the occurrence of postoperative CVSTs away from the operative area remains a pretty rare event and the reasons of this accident remain unknown. Some authors supposed that the intraoperative blood loss or the use of mannitol could have induced dehydration creating an hypercoagulable state [16]. Others supposed that intracranial hypotension secondary to CSF hypovolemia may induce venous dilation resulting in blood stasis into the venous sinus system [17].

Finally, cases of CVST were described after trans-sphenoidal surgery for pituitary adenomas or craniopharyngiomas. In these cases, some authors supposed a concomitant role of the predisposing hormonal condition, perioperative electrolytic alterations and direct manipulation or coagulation of the cavernous sinus wall, which could have triggered the coagulation cascade [18–20].

### Hints, precautions, and therapeutic considerations

Most of the authors agree that in order to help mitigating these potential risk factors, efforts should be made to minimize sinus exposure and manipulation. Drilling around the sinus should be accomplished under constant irrigation, while exposed aspects of the sinus should be protected with moist neurosurgical patties. Retraction of the sinus should generally be avoided or frequently released to prevent intraluminal venous stasis. Finally, in the case of direct sinus injury, repair should be undertaken using nonabsorbable monofilament sutures [8]. We also recommend avoiding the injection of thrombin-based hemostatics or fibrin glue into the sinus lumen as the extent of the hemostatic cascade they trigger cannot be predicted.

The crux of the matter remains the treatment of CVST. The medical management is highly debated, especially the role, safety, and efficacy of anticoagulation. The rational of the anticoagulation would be reducing the risk of sinus thrombosis propagation and the development of venous infarction as it is the mainstay of therapy for spontaneous CVST [3, 8, 9]. However, due to the relatively paucity of related symptoms, there remains significant heterogeneity among clinicians regarding the right management of postoperative CVST as the use of post-operative anticoagulation with a dose higher than prophylactic may expose to the risk of hemorrhage of the surgical bed or at hemorrhagic infarction of any venous infarct area following the occurrence of CVST. Very often, in fact, the diagnosis of CVST is incidental at post-operative neuroimaging and the patients do not complain associated symptoms, and this strongly discourage the use of therapeutic regimen of anticoagulation.

Apra et al. used therapeutic enoxaparin in a majority of patients with lateral CVST finding a statistically significant elevated risk of post-surgical complications compared with patients without anticoagulation (*p* = 0.020) [9].

Moore et al. reported a successful management of postoperative CVST in 5 patients using intravenous heparin transitioned to warfarin for 6 months with low risk of hemorrhagic complications [3].

However, despite it all, concerns about the risk of catastrophic hemorrhages in postoperative period are rampant in surgeons’ minds [12, 16]. Abou-Al-Shaar et al. for example denounced severe hemorrhagic complications attributable to enoxaparin in the immediate postoperative period for treatment of CVST [8]. This has led most of the authors to recently review their therapeutic attitude towards this nosological entity. For instance, Orlev et al. reported a large institutional series along with a literature review regarding transverse/sigmoid sinus thrombosis following suboccipital craniectomy showing a higher rate of early complications; however, most of them resolved without anticoagulation, therefore suggesting to manage these patients conservatively in order to avoid additional hemorrhagic risks [21].

Similarly, Brahimaj et al. reported a unusually higher incidence of CVST after vestibular schwannoma surgery compared with other similar series, but they did not observe any symptomatic patient despite none was treated with anticoagulation [11]. According to their evidence, they did not suggest a routinely use of dedicated vascular imaging such as CT or MR venograms with possible time-of-flight sequences to better delineate the presence of a sinus thrombosis.

However, it is important to note that some of the imaging modalities and in particular the contrast-enhanced MRI, are unable to delineate if the thrombi are flow limiting. A thrombus causing total occlusion versus a flow limiting thrombus may have different clinical consequences.

Therefore, at present, due to the limited data regarding the natural history of post-operative CVST and the absence of guidelines, the rational for treatment and the choice of medications are leaved to the experience of the surgeon [8]. The majority agrees regarding a hydration and hemodilution in the post-operative period and reserving the medications to those patients who had a sinus violation during surgery which required suturing repair. Also, the addition of the aspirin to the anticoagulation with low molecular weight heparin has been proposed by some authors with limited risk [22].

## Conclusions

CVST represents a potential complication of surgical approaches in proximity of dural venous sinuses. CVST is usually segmental and rarely shows a progression. Accordingly, this condition courses uneventfully or with minor symptoms in the vast majority of cases. Thus, the systematic use of post-operative anticoagulants at therapeutic dosage does not seem offer a significant advantage on clinical and radiological outcome of post-operative CVST in the face of the possible hemorrhagic risks.

## Data Availability

Available upon reasonable request.
